# Microfluidic platform for electrophysiological recordings from host-stage hookworm and *Ascaris suum* larvae: A new tool for anthelmintic research

**DOI:** 10.1016/j.ijpddr.2016.08.001

**Published:** 2016-09-15

**Authors:** Janis C. Weeks, William M. Roberts, Kristin J. Robinson, Melissa Keaney, Jon J. Vermeire, Joseph F. Urban, Shawn R. Lockery, John M. Hawdon

**Affiliations:** a Institute of Neuroscience and African Studies Program, University of Oregon, 1254 University of Oregon, Eugene, OR 97403-1254, USA; b Institute of Neuroscience, University of Oregon, 1254 University of Oregon, Eugene, OR 97403-1254, USA; c Department of Microbiology, Immunology, and Tropical Medicine, School of Medicine and Health Sciences, The George Washington University, Washington, DC 20037, USA; d Center for Discovery and Innovation in Parasitic Diseases, Dept. of Pathology and Laboratory Medicine, UC, San Francisco, USA; e US Department of Agriculture, Agricultural Research Service, Beltsville Human Nutrition Research Center, Diet, Genomic and Immunology Laboratory, Beltsville, MD, USA

**Keywords:** Anthelmintic, Electropharyngeogram, *Ancylostoma*, *Ascaris*, Microfluidic, Electrophysiology, Hookworm, Ivermectin, ScreenChip, Serotonin

## Abstract

The screening of candidate compounds and natural products for anthelmintic activity is important for discovering new drugs against human and animal parasites. We previously validated in *Caenorhabditis elegans* a microfluidic device (‘chip’) that records non-invasively the tiny electrophysiological signals generated by rhythmic contraction (pumping) of the worm's pharynx. These electropharyngeograms (EPGs) are recorded simultaneously from multiple worms per chip, providing a medium-throughput readout of muscular and neural activity that is especially useful for compounds targeting neurotransmitter receptors and ion channels. Microfluidic technologies have transformed *C. elegans* research and the goal of the current study was to validate hookworm and *Ascaris suum* host-stage larvae in the microfluidic EPG platform. *Ancylostoma ceylanicum* and *A. caninum* infective L3s (iL3s) that had been activated *in vitro* generally produced erratic EPG activity under the conditions tested. In contrast, *A. ceylanicum* L4s recovered from hamsters exhibited robust, sustained EPG activity, consisting of three waveforms: (1) conventional pumps as seen in other nematodes; (2) rapid voltage deflections, associated with irregular contractions of the esophagus and openings of the esophogeal-intestinal valve (termed a ‘flutter’); and (3) hybrid waveforms, which we classified as pumps. For data analysis, pumps and flutters were combined and termed EPG ‘events.’ EPG waveform identification and analysis were performed semi-automatically using custom-designed software. The neuromodulator serotonin (5-hydroxytryptamine; 5HT) increased EPG event frequency in *A. ceylanicum* L4s at an optimal concentration of 0.5 mM. The anthelmintic drug ivermectin (IVM) inhibited EPG activity in a concentration-dependent manner. EPGs from *A. suum* L3s recovered from pig lungs exhibited robust pharyngeal pumping in 1 mM 5HT, which was inhibited by IVM. These experiments validate the use of *A. ceylanicum* L4s and *A. suum* L3s with the microfluidic EPG platform, providing a new tool for screening anthelmintic candidates or investigating parasitic nematode feeding behavior.

## Highlights

• Pharyngeal pumping in nematodes generates an electropharyngeogram (EPG).
• The EPG provides a readout of the electrical activity of neurons and muscles.
• A microfluidic platform for recording EPGs was validated in parasitic nematodes.
• EPG activity and drug responses were characterized in host-stage larvae.
• Microfluidic EPG recordings provide a powerful new tool for anthelmintic research.

## Graphical abstract

**Figure:**
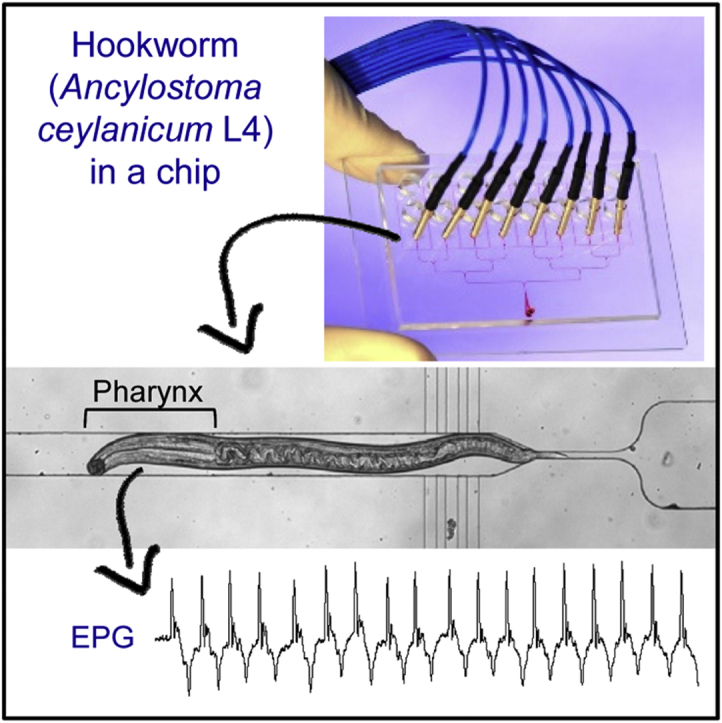

## Abbreviations

5HT: 5-hydroxytryptamine (serotonin)
aL3: activated L3
CaS: calf serum
CF_50_: time at which 50% of the total number of EPG events had occurred after switching perfusate
CS: canine serum
E spike (in EPG recording): onset of muscle contraction during a pump
EI: esophogeal-intestinal
EPG: electropharyngeogram
FITC-BSA: bovine serum albumin–fluorescein isothiocyanate conjugate
FF: flutter fraction
Glu-Cl: glutamate-gated chloride channel
GSM: S-methyl-glutathione
HS: human serum
iL3: infective L3
IPSP: inhibitory postsynaptic potential
IVM: ivermectin
M9: M9 buffer
PBS-ps: PBS containing 100 U penicillin and 100 μg/ml streptomycin
PC: polycarbonate
PDMS: polydimethylsiloxane
PE: polyethylene
RPMI-c: complete RPMI medium
R spike (in EPG recording): muscle relaxation during a pump
SNR: signal-to-noise ratio
STH: soil-transmitted helminth

## 1 Introduction

Intestinal parasites cause considerable disease burdens in humans and other animals. Soil-transmitted helminth (STH) infections are concentrated in sub-Saharan Africa, the Americas, China and East Asia, where over a billion people carry these parasites. Infections with hookworm (*Ancylostoma* spp. and *Necator americanus*), roundworm (*Ascaris lumbricoides*) and whipworm (*Trichuris trichuria*) cause physical and cognitive stunting in children, and chronic ill health and impaired productivity in adults (Bethony et al., 2006, Brooker, 2010). Likewise, gastrointestinal nematode infections in livestock cause poor productivity and economic losses in both high- and low-income nations (Pfukenyi and Mukaratirwa, 2013, van der Voort et al., 2016).

Current anthelmintic (anti-worm) drugs have limitations. For example, some parasites (e.g., human whipworm, *T. trichuria)*, are relatively insensitive to all available anthelmintic drugs (Keiser and Utzinger, 2008). Additionally, increasing drug resistance in parasites is weakening the effectiveness of current anthelmintics, especially in veterinary medicine (Kaplan, 2004, Coles et al., 2006). Drugs are failing in both livestock and companion animals (Wolstenholme et al., 2015). In humans, reports of reduced anthelmintic efficacy are not widespread but, given the nature of natural selection, are expected to increase (Vercruysse et al., 2011). It is widely agreed that new anthelmintic treatments are urgently needed and that the current drug development pipeline is inadequate (Geary et al., 2015).

Our research addresses two key aspects of the search for new anthelmintics: (1) the nature of the physiological readout used to detect anthelmintic bioactivity and (2) the nematode species used for screening. When screening candidate molecules on cultured worms, typical endpoints include developmental arrest, impaired motility or death (Geary et al., 2015). These phenotypes are easily scored but provide little insight into underlying mechanisms. For high-throughput screening, the free-living nematode *Caenorhabditis elegans* offers convenience, low cost and molecular-genetic tools (Holden-Dye and Walker, 2014, Burns et al., 2015), but has yet to produce a commercial product (Geary et al., 2015). Hits identified in *C. elegans* must subsequently be tested on the targeted parasitic species or close relatives. Alternatively, parasitic nematodes can be used in primary screens (e.g., Bulman et al., 2015), at higher cost and lower throughput than *C. elegans*, but with more direct relevance to targeted species.

Many current anthelmintic drugs act on proteins involved in electrical signaling—neurotransmitter receptors and ion channels—and these molecules remain valuable targets for anthelmintic drug development (Wolstenholme, 2011, Taman and Azab, 2014). Therefore, a screening method that reads out electrophysiological function could help prioritize and characterize hits when seeking new anthelmintics, and provide more mechanistic insight than nonspecific phenotypes such as death. We addressed this need by integrating the fields of microfluidics (the precise control of fluids and samples at sub-millimeter scale) and electrophysiology to develop a device (‘chip’) that noninvasively records, in real time and medium throughput, the tiny electrical signals emitted by nematode muscles and neurons (Lockery et al., 2012). This device contributes to an ongoing revolution in *C. elegans* research fueled by microfluidic technologies (Bakhtina and Korvink, 2014). Our device records electropharyngeograms (EPGs) from eight worms simultaneously during exposure to control or test substances. EPG recordings reveal electrical activity of muscles and neurons of the pharynx (sometimes termed the esophagus), the muscular pump used for feeding (Raizen and Avery, 1994, Trojanowski et al., 2016). In *C. elegans*, pharyngeal pumping draws bacteria into the digestive tract, whereas intestinal parasites ingest host digesta, blood and/or tissue (Wells, 1931, Kalkofen, 1970, Khuroo, 1996, Avery and You, 2012). Pharyngeal pumping frequency is often used to assess nematodes' physiological status (Hughes et al., 2011), but is typically counted only for brief (10–60 s) periods, and by eye, which can be inaccurate. In contrast, EPG recordings can provide millisecond-resolution data on thousands of pumps (see 2.9), providing exceptional statistical power. A microfluidic chip that records EPGs from single worms has also been developed in the laboratory of Prof. Lindy Holden-Dye (C. C. Hu et al., 2013, Y. Hu et al., 2013).

We have extensively validated the 8-channel microfluidic EPG platform in *C. elegans,* including characterization of the dose-dependent inhibition of pumping by anthelmintic drugs and distinguishing wild type from drug-resistant worms (Lockery et al., 2012; Weeks et al., in preparation). A one-channel version of this chip is now commercially available (‘ScreenChip’; http://nemametrix.com). The goal of the present study was to adapt the technology and software for use with parasitic nematodes that impact human and animal health. Specifically, we optimized methods for two classes of human STHs: (1) hookworms, including *Ancylostoma ceylanicum,* a significant human parasite in SE Asia (Traub, 2013); and (2) *Ascaris suum*, a zoonotic model for the human parasite, *A. lumbricoides,* which may be the same species (Leles et al., 2012). Our experiments demonstrate successful adaptation of the microfluidic EPG platform for use with host-stage STH larvae, providing a new tool for anthelmintic research and investigations of nematode feeding behavior.

## 2 Materials and methods

### 2.1 Animal care

Animals were housed and treated in accordance with institutional animal care and use committee guidelines at The George Washington University (protocol A270), USDA/ARS (protocol 13-019) and UC San Francisco (protocol AN098756-02).

### 2.2 *In vitro* activation of infective L3s

The western Maryland (wmd) strain of *A. caninum* (US National Parasite Collection No. 106970) was maintained in beagles, and infective L3 worms (iL3s) were harvested from charcoal cultures of dog feces (Krepp et al., 2011). An Indian strain of *A. ceylanicum* (USNPC No. 102954) was maintained in Syrian golden hamsters (Garside and Behnke, 1989) and iL3 were stored in BU buffer (Hawdon and Schad, 1991) at room temperature for up to 5 wk until use. iL3 of both species were activated under host-like conditions as described previously (Hawdon et al., 1999). Briefly, ∼250 decontaminated iL3 were incubated at 37 °C, 5% CO_2_, for 24 h in 96-well tissue culture plates containing 0.1 ml RPMI-1640 tissue culture medium (Mediatech, Inc. 10-041-CV, Manassas, VA) with 25 mM HEPES (pH 7.0), and supplemented with 100 U penicillin, 100 μg/ml streptomycin (penicillin-streptomycin solution, Global Cell Solutions, North Garden, VA) and 100 μg/ml gentamycin (Sparhawk Laboratories, Lenexa, KS). This medium is designated RPMI-complete (RPMI-c). iL3 were activated by adding 15 mM S-methyl-glutathione (GSM, Sigma M4139) and 10% (v/v) of human serum (HS; Sigma H4522, St. Louis, MO) or a <10 kDa ultrafiltrate of canine serum (CS; recovered from whole blood collected from post-infection dogs and stored at −20 °C; Hawdon et al., 1995) to the RPMI-c. After 24 h incubation, worms were returned to RPMI-c. Feeding, an indicator of successful activation (Hawdon and Schad, 1990), was assayed in *A. caninum* by incubating worms for 2–3 h in 5 mg/ml of FITC-BSA (Sigma A9771) in RPMI-c. Larvae with FITC-BSA in the intestinal tract were used for EPG recordings.

### 2.3 Collecting *A. ceylanicum* L4s from hamsters

To obtain developing parasitic stages, 4- to 5-week-old Syrian golden hamsters were inoculated orally using a feeding needle with ∼2000 *A. ceylanicum* iL3s. At ∼72 h post-infection, hamsters were killed by CO_2_ inhalation, the small intestine was removed, split longitudinally, and placed in warm PBS with 100 U penicillin and 100 μg/ml streptomycin (PBS-ps) at 37 °C to allow worms to release from the intestine. L4s were individually selected based on size and the characteristic buccal capsule. L4s were rinsed twice in warm PBS-ps and incubated in RPMI-c at 37 °C until use.

### 2.4 Collecting *A. suum* L3s from swine

The preparation of infective *A. suum* eggs and inoculation of two pigs from the Beltsville Swine Herd were completed as described previously (Urban et al., 2013). Seven days after inoculation with 15,000 infective eggs, the lungs were removed and mixed with an equal volume of 37 °C normal saline in a Waring blender container with rotating cutting blades, and homogenized for approximately 20 s to produce a tissue suspension of fragments of ∼0.5 cm^3^. Host-stage L3s were isolated from the lung tissue using an agar-gel method (Urban et al., 2013), transferred to RPMI-c and kept in a 37 °C, 5% CO_2_, incubator until use. For EPG recordings, 10% (v/v) calf serum (CaS; Lonza BioWhittaker 14-401F, Walkersville, MD, USA) was added to RPMI-c.

### 2.5 Microfluidic EPG devices

**Fig. 1:**
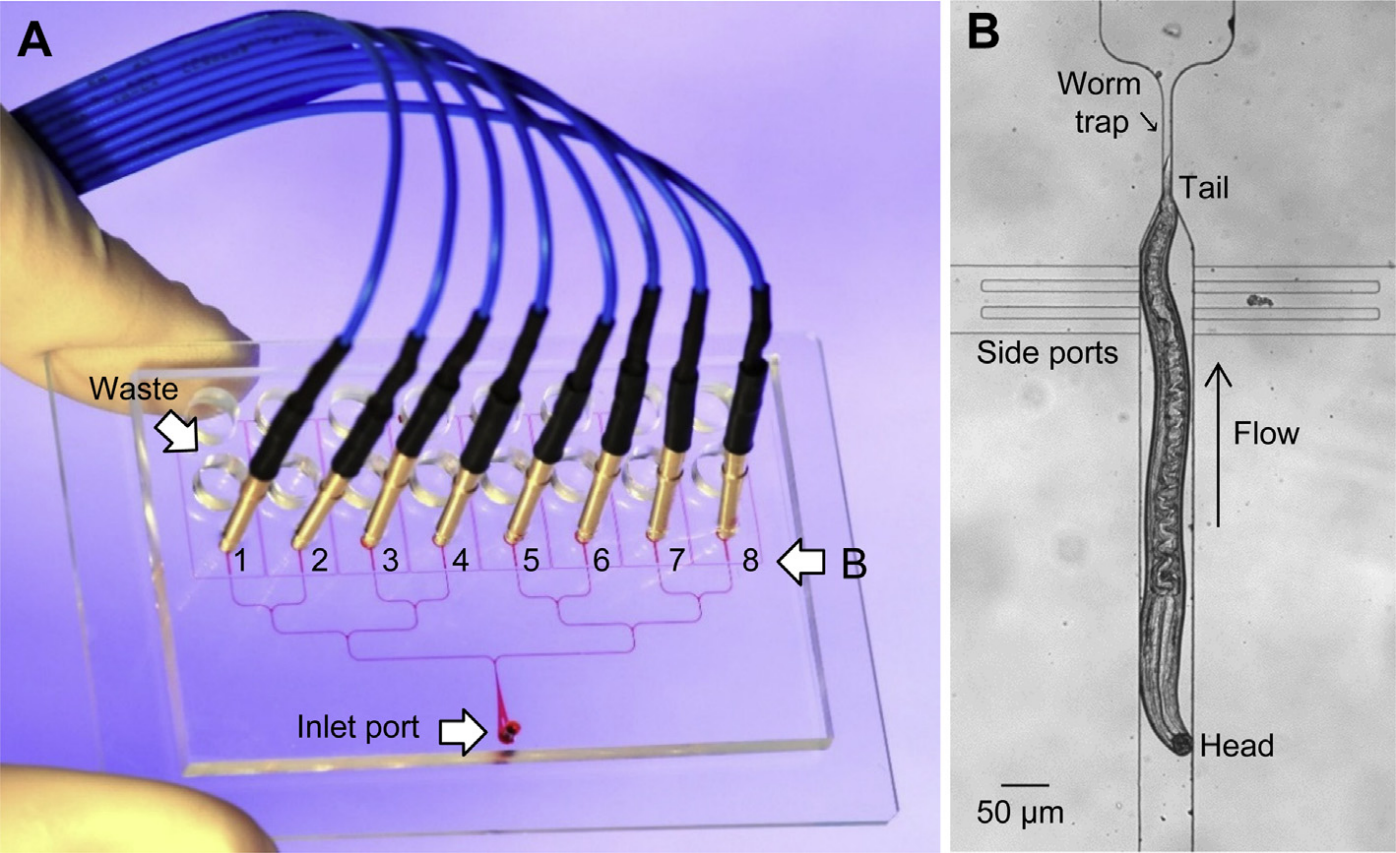
Microfluidic EPG recording device. A. Channels were filled with a dye to aid visualization in this image. Dimensions of the glass substrate of the chip was 5.08 × 7.62 cm, with PDMS layer above. Worms were loaded into the inlet port (arrow) and distributed via a branching network into narrowed channel segments (‘worm traps’; see B) located in each of the 8 recording modules. An electrode (blue wire) was inserted distal to each worm trap and a hollow metal electrode (not shown) was inserted into the inlet port to deliver perfusate and serve as a common electrical reference. After flowing past worms, perfusate collected in a row of waste reservoirs (arrow). Expanded region shown in B is indicated (arrow). B. Enlarged view of a single recording module, with an *A. ceylanicum* L4 positioned tail-first in the worm trap. (For interpretation of the references to colour in this figure legend, the reader is referred to the web version of this article.)

Devices (‘chips’) were fabricated at the University of Oregon using standard soft lithographic methods (Xia et al., 1996, Xia and Whitesides, 1998). Each chip had eight recording modules (see Fig. 1A). Channel dimensions in the PDMS (polydimethylsiloxane) layer were modified to optimize EPG recordings from different nematode species and stages. It was not possible to developmentally synchronize parasitic worms to the same extent as *C. elegans*, so they had more size variation. Variation in length was not an important factor whereas worm diameter was, as it affects the seal resistance and therefore the signal-to-noise ratio (SNR; Lockery et al., 2012). For *A. ceylanicum* L4s, chips had a channel height of 45 or 55 μm and width of 60 μm. For *A. suum* L3s, channel height was 75 μm and width was 60 μm. For aL3s, which were smaller, we used a new design in which channel width tapered gradually from 60 μm to 30 or 20 μm. Height of the tapered channels was 25 or 35 μm. Depending on diameter, aL3s lodged at different distances along the tapered channel. In all designs, the ‘worm trap’ (see Fig. 1B) was 13 μm wide, too narrow to permit worms to pass.

### 2.6 Loading and recording from EPG chips

Chips were preloaded with M9 buffer (Stiernagle, 2006) containing 0.01% Tween (Fisher Scientific BP337-500); Tween facilitated propelling the long, coiling larvae along the network of channels that distribute worms into recording channels (see Fig. 1A). This solution also avoided short-circuiting of electrical signals by RPMI-c left on the surface of the PDMS during loading. Worms were gently propelled into position by applying pressure into the inlet port via a syringe and polyethylene (PE) tubing (PE-BPE-T25, Instech Laboratories, Inc., Plymouth Meeting, PA, USA) filled with the M9-Tween solution. Worms lodged either head- or tail-first in the recording modules, which determined signal polarity (Lockery et al., 2012). By convention (Raizen and Avery, 1994), EPG traces in the Figures are displayed with the ‘E’ spike of the waveform (indicating onset of muscle contraction) upward and the ‘R’ spike (muscle relaxation) downward. During the E spike, the extracellular voltage at the head is positive relative to the tail (Raizen and Avery, 1994).

The loaded chip was positioned on a stereomicroscope (Leica MZ16 or Olympus VMZ) or inverted microscope (Nikon TMS or Zeiss Axio Observer A1). Polycarbonate tubing (PC; CTPC450-900-5, Paradigm Optics, Vancouver, WA, USA) was inserted into the inlet port via a short length of 1.5 mm diameter stainless steel tubing (New England Small Tube, Litchfield, NH, USA) that also served as the reference electrode. The other end of the tubing led to a syringe on a syringe pump (Harvard Apparatus PHD 2000; Holliston, MA). The PC tubing was connected to the reference electrode and syringe by short lengths of PE tubing. Solutions were perfused through the chip at 6 μl/min and solution changes were effected by manually switching the tubing to a different syringe on the pump. The resultant electrical artifact was masked in the Figures. The time between switching syringes and the new perfusate reaching worms was <60 s. A silver metal electrode was inserted into a port distal to each worm trap, which led to differential amplifiers that measured voltage relative to the reference electrode. Chips were typically maintained between 34 and 38 °C via a heater mounted on the aluminum dock holding the chip, consisting of a 1 Ω power resistor affixed to the dock by its aluminum heat sink. Heat sink compound was used to facilitate heat transfer between the aluminum and glass contacts, and foam insulation was used to reduce heat loss. The heater was powered from a rheostat-controlled 5 V DC power supply. Temperature was monitored by a miniature thermocouple inserted between the PDMS layer and glass substrate of the chip, which led to a digital thermometer (Signstek 6802II, Wilmington, DE, USA). The rheostat was adjusted manually to maintain temperature.

### 2.7 Drug solutions

Stocks of serotonin creatine sulfate monohydrate (Sigma H7752; St. Louis, MO) were prepared in M9 buffer at 40 mM and held in small aliquots at −20 °C until use. Each day of an experiment, a fresh aliquot was thawed and diluted to the desired concentration. Working solutions of 5HT were used within 70 min of preparation. Stocks of ivermectin (10 mM; Sigma 8898) were prepared in 100% dimethyl sulfoxide (DMSO; Fisher D-136; Fair Lawn, NJ), held at −20 °C until use and diluted to the working concentration each day.

### 2.8 Electrophysiological and video recordings

EPG signals were acquired as described in Lockery et al. (2012) with few modifications. AC amplifiers (A-M Systems model 1700 or 3500; Carlsborg, WA) had low-and high-frequency cut-offs of 1 and 500 Hz, respectively, and a 60 Hz notch filter. Signals were digitized at 2.5 kHz/channel and displayed in Spike2 software (version 7.06a, Cambridge Electronic Design). Data were acquired continuously during each experiment. An additional channel was used as a keystroke-controlled event marker. Because the amplitude of EPG signals varied (see Table 1) voltage scales were not included in figures, and traces were scaled to have similar peak-to-peak amplitudes.

Videos of *A. ceylanicum* pharyngeal behavior were acquired using (1) a smartphone video application to film a video monitor displaying the worm (30 frames/s) or (2) a CMOS camera (DFK 23UM021; The Imaging Source, Charlotte, NC, USA) attached to the microscope (up to 80 frames/s). Video and EPG recordings were synchronized using Igor Pro (WaveMetrics, Lake Oswego, OR, USA). Audio for Fig. 4 was generated by modulating the volume of broad-band, low-frequency noise by the voltage amplitude of the EPG trace.

### 2.9 EPG data analysis

A 60 min EPG recording of a single larva generating EPG events at 1 Hz (Fig. 5A) contains ∼3600 events, far beyond the ability of an investigator to analyze by hand. Accordingly, we used an automated spike recognition system modified from that in Lockery et al. (2012). Recordings acquired in Spike2 were down-sampled to 500 Hz, imported into Igor Pro as text files, and analyzed using a pump-recognition algorithm developed for EPG recordings from *C. elegans*, which will be published elsewhere (JC Weeks, KJ Robinson, SR Lockery and WM Roberts, unpublished data). Parasite movements within recording modules were more vigorous than for *C. elegans*, causing EPG waveforms to vary in amplitude and shape between worms and in the same worm over time. This variability provided greater challenges for automated pump identification than EPG recordings made by transecting *C. elegans* and sucking the exposed pharynx into a pipette (forming a seal with relatively high electrical resistance and well-defined geometry), the conditions used by Dillon et al. (2009) for their ‘AutoEPG’ pump-recognition program. To accommodate EPG variability, the algorithm used in the present study compensated for changes in EPG amplitude during the recording and automatically optimized recognition of each worm's EPG waveforms. Attributes including the time, duration and amplitude of each pump were collected automatically by the software.

Because *A. ceylanicum* L4s produced a second EPG waveform that we have termed a flutter, and a hybrid waveform with features of both pumps and flutters (see 3.3), the *C. elegans* pump-recognition algorithm was modified to identify and count clusters of three or more closely-spaced pairs of positive and negative peaks as a single flutter. Hybrid waveforms having only two closely-spaced pairs of positive and negative peaks, or multiple unpaired peaks, were counted as one ‘pump’.

To test the accuracy of pump and flutter identifications obtained by the automated analysis versus those made by human observers, two individuals familiar with EPG recordings (JCW and WMR) scored 1 min of baseline data (beginning at *t* = −12 min before switching solutions) from two *A. ceylanicum* L4s selected randomly from each of the 12 experimental groups in Fig. 5, Fig. 6, Fig. 7 (i.e., 24 min of EPG data from 24 different worms; each worm scored by one observer). Scorers were blinded to the automated identification of the waveforms. In the 24 min of data analyzed, the scorers identified 1836 events (pumps + flutters; mean event frequency = 1.27 Hz) whereas the automated detection algorithm identified 1655 events (mean event frequency = 1.15 Hz). Thus, the algorithm may have underestimated mean event frequency by ∼10%. Combining all events identified by either the algorithm or the scorers, and assuming that the scorers made correct identifications, there was 86% concordance (true positives), 2% false positives, and 12% false negatives. The primary causes of mismatches were: (1) the EPG signal became too small to distinguish from background noise (poor SNR), or (2) an event was obscured by a brief electrical artifact of unknown cause. The concordance between human scorers and the algorithm in identifying waveforms as pumps vs. flutters was 84%; because pumps and flutters formed a continuum (Fig. 3B), identifying a waveform as one or the other (by algorithm or human scorer) was sometimes arbitrary so this discordance was unsurprising.

EPG recordings were rejected if the signal was deemed too noisy for reliable identification of pumps by a human observer or if the worm turned around in the microfluidic channel during the recording. The remaining recordings were passed to the automated analysis program, which recorded the time, duration and amplitude of each pump or flutter. For each recording, the baseline event frequency (*f*_*baseline*_) was determined by counting the number of events during the 10 min immediately before switching the perfusion source (−12 min < *t* < −2 min). To improve consistency of the results, worms with low baseline event frequencies (*f*_*baseline*_ < 0.45 Hz) were eliminated from further analysis. Eliminating worms with low *f*_*baseline*_ also eliminated the large variance that is introduced into the calculation of normalized event frequency (see below) when the denominator is close to zero. Approximately 10% of worms were excluded by this criterion.

The graphs in Fig. 5, Fig. 6, Fig. 7 show mean event frequencies averaged across worms (i.e., ensemble-averaged frequencies), with shading drawn to indicate ± 1 S.E.M., where *n* is the number of worms in the ensemble. The event frequency, *f*_*event*_(*t*), was first calculated separately for each worm by binning the time axis (1 s bin width), counting all events (pumps + flutters) in each bin, and smoothing the result using a Gaussian weighted sliding window with σ=30 s. The smoothed event frequency vs. time curves were then averaged across worms to determine the mean and S.E.M. at each time point.

Normalized event frequency was calculated as *f*_*event*_(*t*)*/f*_*baseline*_. We also computed the ‘flutter fraction’ (FF), defined as the proportion of events that were flutters:

$$\mathrm{FF}\left(t\right)=f_{flutter}\left(t\right)/\left(f_{flutter}\left(t\right)+f_{pump}\left(t\right)\right)$$

where the pump frequency, *f*_*pump*_(*t*), and flutter frequency, *f*_*flutter*_(*t*), were computed separately and smoothed as described above for *f*_*event*_(*t*) before taking the ratio. To reduce the effects of baseline variability between worms, each worm's baseline flutter fraction between −12 and −2 min was subtracted before averaging across worms.

To compare statistically the time required for drug effects, we computed the cumulative fraction (CF) of EPG events that occurred following drug onset, with *CF*_50_ defined as the time at which 50% of the total number of events occurred during the 45 or 60 min observation period after drug onset. Drugs that rapidly blocked pumping thus produced small *CF*_50_ values; drugs that had no effect on pumping had *CF*_50_ values equal to one half of the post-drug observation time. This definition was used because EPG activity of individual worms often stopped and restarted multiple times after drug addition, causing ambiguity in measures such as the time for the mean event frequency to fall by 50%. The cumulative fraction method avoids this ambiguity because it rises monotonically with time.

## 3 Results and discussion

### 3.1 EPG recording method for parasitic larvae

Fig. 1A shows the design of the 8-channel microfluidic EPG chip used in these experiments, with channel dimensions optimized for the species and stages studied (see 2.5). Each worm was positioned in a recording module (Fig. 1B), randomly oriented either head- or tail-first, which determines signal polarity (Lockery et al., 2012). All EPG recordings shown here are displayed with the E spike (see 3.2) directed upward.

### 3.2 EPG recordings from *A. ceylanicum* and *A. caninum* iL3s activated *in vitro*

In *Ancyclostoma* spp., eggs in the feces of infected hosts are deposited onto soil and develop into infective L3s (iL3s), which do not feed. After entering a suitable host, iL3s shed their enveloping cuticle (‘exsheathment’) and commence feeding. Exposure to suitable conditions *in vitro* (e.g., serum components, glutathione analogs, elevated temperature and CO_2_) can ‘activate’ iL3s to transform into host-stage L3s and initiate feeding (e.g., Hawdon and Schad, 1990, Tritten et al., 2012). Activated L3s are henceforth termed aL3s. This method for obtaining host-stage larvae is simpler and less expensive than sacrificing mammals to obtain worms.

We tested whether aL3s of *A. ceylanicum* and *A. caninum* were suitable subjects for screening compounds using the EPG platform, with the performance criteria being the generation of: (1) recognizable EPG waveforms (confirmed by simultaneous visual observation of pharyngeal pumping movements) with good SNR and (2) EPG activity that continued at a relatively regular frequency for at least 60 min while worms were in chips. These criteria were based on *C*. *elegans* adults, which pump for hours at ∼4–5 Hz in the presence of 5HT, permitting sensitive detection of anthelmintic bioactivity (Lockery et al., 2012 and unpublished data). EPGs were recorded from *A. ceylanicum* and *A. caninum* aL3s in RPMI-c or M9 buffer, with various additives (5HT, GSM and blood sera) that stimulate feeding (Roche et al., 1971, Hawdon and Schad, 1990). We tested M9 because we use it routinely for EPG recordings in *C. elegans* (Lockery et al., 2012).

**Fig. 2:**
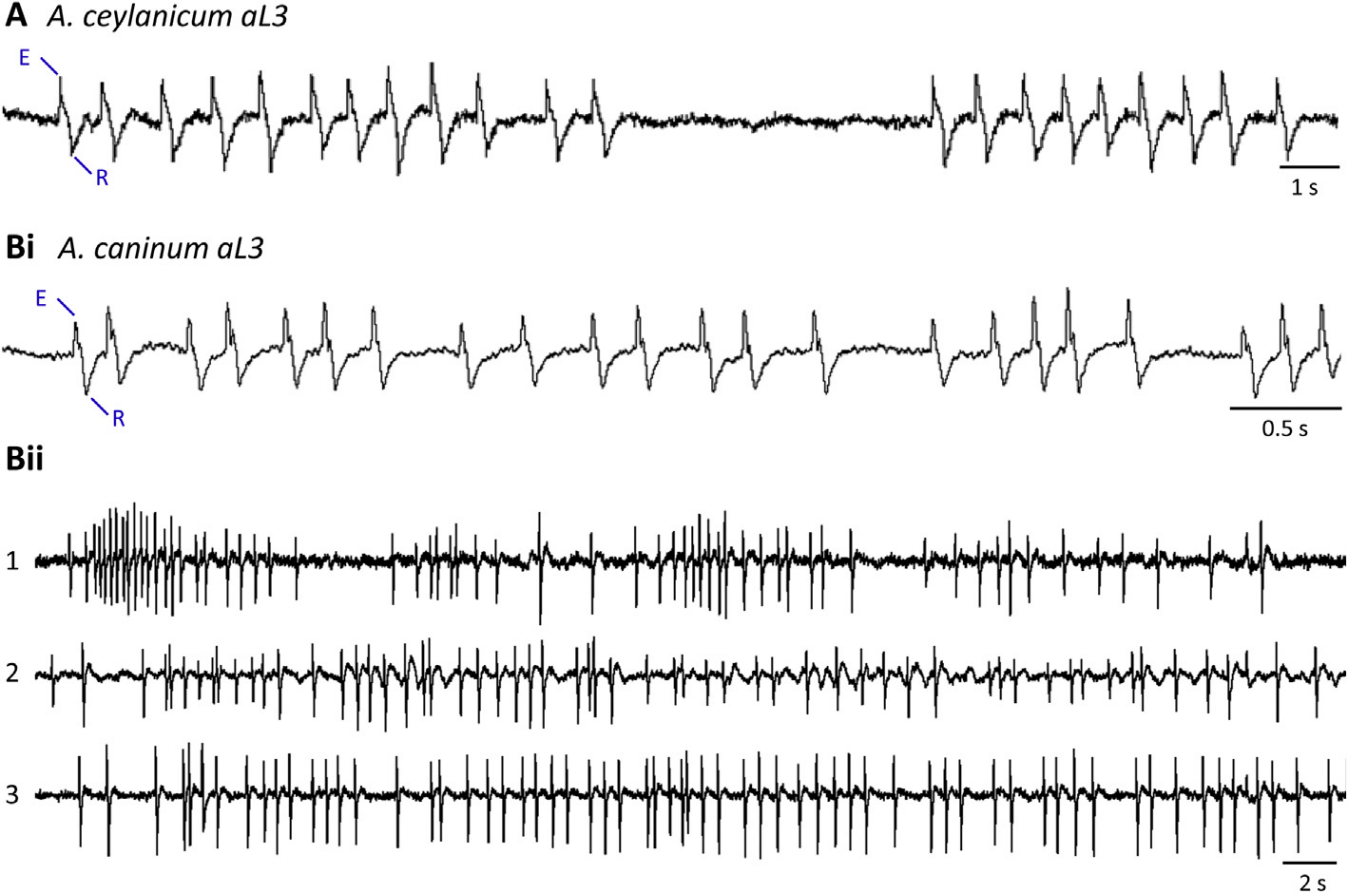
Microfluidic EPG recordings from activated L3 (aL3) hookworms. A. *A. ceylanicum* aL3s, in M9 with 15% HS and 10 mM 5HT, 28 °C. E and R spikes in the pump waveform are marked in A and Bi. B. *A. caninum* aL3s. i. In M9 with 1 mM 5HT, 38 °C. ii. Three worms in one chip (numbered 1 to 3; each trace from a different worm), in M9 with 5 mM 5HT, 42 °C. All worms were recorded 1 d after *in vitro* activation.

Pharyngeal pumping and the expected EPG waveforms were observed in aL3s of both hookworm species (Fig. 2). *A. ceylanicum* aL3 recordings were obtained primarily at room temperature whereas, in all other experiments, worms were maintained closer to host temperature. The characteristic pump waveform reported in many nematode species (e.g., Raizen and Avery, 1994, Sheriff et al., 2002, Tahseen et al., 2003, Hu et al., 2014) was apparent, with pronounced E and R spikes marking the excitation (contraction) and repolarization, respectively, of pharyngeal muscle. Most aL3s produced 1 or more clearly-recognizable pump waveforms during EPG recordings: 80% (41 of 51) of *A. ceylanicum* and 79% (46 of 58) of *A. caninum*. However, we did not successfully identify conditions that stimulated sustained pumping. For most (51 of 58) *A. caninum* EPG recordings, the worms were first incubated in FITC-BSA and selected for strong intestinal labeling, indicating commencement of feeding (Hawdon and Schad, 1990). Occasionally, aL3s exhibited regular pumping (Fig. 2Bii) but sustained activity was an exception and pumping was normally infrequent and erratic. The inclusion of serum and 5HT in RPMI-c, and recording at warmer temperatures, seemed the most effective in stimulating pumping.

Table 1 presents the amplitude and duration of pharyngeal pumping EPG waveforms. The two species of aL3s had the smallest pump amplitudes. Pump amplitude is affected by variables including the tightness of fit of a worm in its recording module, the strength of each contraction and the size of the pharynx (larger muscles emit more current). The aL3s were the smallest worms from which we recorded (see 2.5), which may account for their smaller EPG amplitudes. Despite the smaller signals, the SNR was satisfactory (Fig. 2). Regarding pump duration, Table 1 shows that *A. ceylanicum* aL3s had significantly longer pump durations than *A. caninum* aL3s, which perhaps resulted from the former recordings being obtained at lower temperature.

In summary, *A. ceylanicum* and *A. caninum* aL3s did not meet our performance criteria for assaying anthelmintic bioactivity in EPG chips (criterion 2). Pardoxically, EPG activity was weak even in aL3s with strong FITC-BSA labeling, indicating that the worms had been feeding in culture. Possibly, FITC-BSA labeling resulted from a relatively low level of pumping, which fell below our performance criterion. Or, pumping may have been robust in culture but inhibited when aL3s were tested in chips; however, because *A. ceylanicum* L4s pump robustly in chips (see 3.3), any such inhibition was not a general phenomenon. Finally, other investigators have reported that larvae activated *in vitro* may differ from their counterparts developing *in vivo* (Joachim et al., 2001, Lin et al., 2013). This potential issue could be addressed by testing host-stage L3s obtained from hamsters. On the positive side, ∼80% of aL3s produced at least some pumping in EPG chips and further attempts to optimize conditions are warranted. However, in the present study, we set aside aL3s and focused on *A. ceylanicum* L4s obtained from hamsters.

### 3.3 EPG recordings in *A. ceylanicum* L4s

**Fig. 3:**
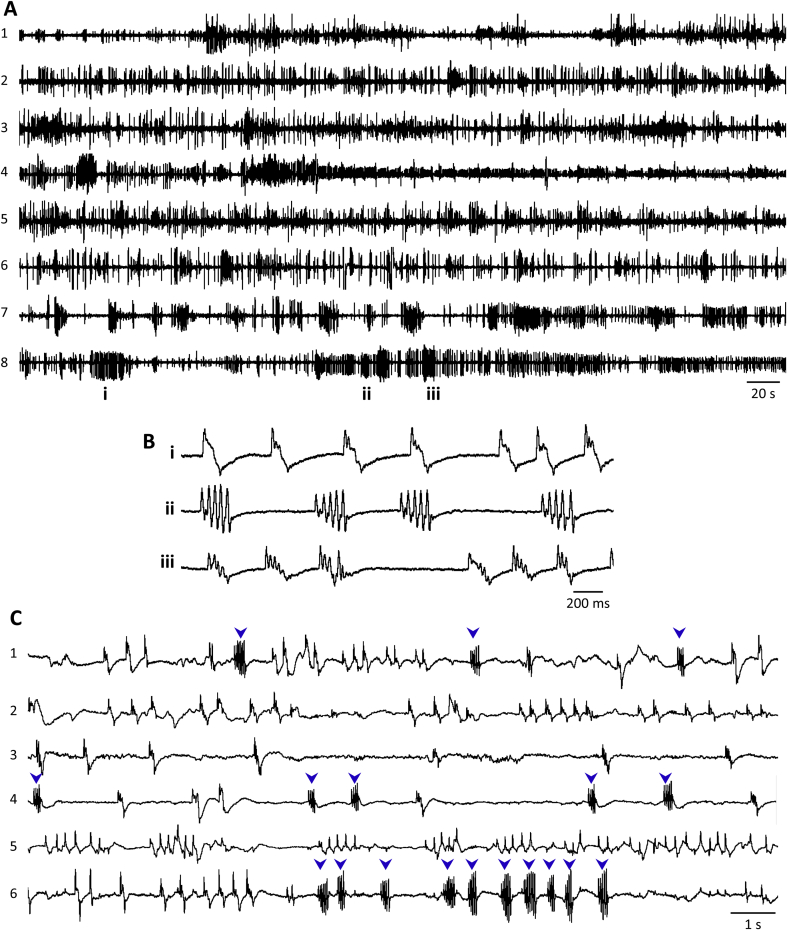
EPG recordings from *A. ceylanicum* L4s. A. Eight worms recorded simultaneously in one chip (numbered 1 to 8; each trace from a different worm), showing robust EPG activity. The times indicated (i, ii, iii) for worm 8 correspond to the traces in B. B. EPG waveforms excerpted from worm 8 in A. i, characteristic pump waveform. ii, waveform that we termed a ‘flutter,’ characterized by rapid voltage deflections. iii, hybrid waveform with features of both pumps and flutters. C. EPG recordings from six *A. ceylanicum* L4s in a different chip (numbered 1 to 6; each trace from a different worm), showing typical diversity in EPG waveforms; flutters marked by blue arrowheads. Recordings made in RPMI-c with 20% CS, 36–38 °C. (For interpretation of the references to colour in this figure legend, the reader is referred to the web version of this article.)

Fig. 3A shows EPG activity in *A. ceylanicum* L4s removed from the small intestine of a hamster ∼72 h post-infection (see 2.3). Unlike aL3s, these worms exhibited sustained activity for prolonged periods of time in RPMI-c with 20% CS, even in the absence of 5HT. In contrast to the clocklike pumping at ∼4–5 Hz in *C. elegans* treated with 5HT (Lockery et al., 2012), EPG activity in *A. ceylanicum* L4s was more bout-like, with an average frequency of ∼1 Hz (see 3.4). Pump waveforms in *A. ceylanicum* L4s resembled those in other nematodes. Table 1 shows EPG pump amplitude and duration values for these worms. Mean amplitude was the largest of any nematodes tested in this study and pump duration was the longest of the nematodes tested at warmer temperatures.

Unexpectedly, EPGs from *A. celanicum* L4s included, in addition to pump waveforms (Fig. 3Bi), a zig-zag-shaped waveform (Fig. 3Bii) and a waveform that appeared to be a hybrid of the first two (Fig. 3Biii). Based on video analysis of esophageal (pharyngeal) behaviors (see 3.4), we named the zig-zag-shaped waveforms ‘flutters.’ Fig. 3C illustrates typical patterns of EPG activity in six different worms; flutters occurred singly (worm 1) or in bouts (worms 4, 6). The initial voltage deflection in a flutter had the same polarity as the E spike of a pump. The number of deflections per flutter ranged from 3 to 8, with 4–6 being most common, and flutters had longer durations than pumps. The hybrid waveform resembled a pump with flutter-like deflections between the E and R spikes. In fact, pump and hybrid waveforms appeared as a continuum, as seen by comparing Fig. 3Bi and Biii. In Fig. 3Bi, all but one of the pumps had small, flutter-like deflections between the E and R spikes, which were qualitatively similar to the deflections in hybrid waveforms (Fig. 3Biii) but of smaller amplitude. A consistent feature of hybrid waveforms was that the peak amplitude of upward-going deflections became smaller over the course of the waveform whereas, during flutters, the amplitude of upward-going deflections remained the same or increased. Because the hybrid waveforms always had obvious E and R spikes, and corresponded to pharyngeal pumps in video analysis (see 3.4), they were catgorized as pumps during our analysis (see 2.9).

Remarkably, over 50 years ago, Roche et al. (1962) reported similar waveforms during EPG recordings (termed ‘electroesophagrams’ by these investigators) from *A. caninum* adults. ‘A waves’ were recognizable as pump waveforms and confirmed as such by visual observation. ‘B waves’ appeared identical to what we here term flutters. Hybrid waveforms were not mentioned. Roche et al. reported that B waves corresponded with “ … uncoordinated inefficient movement of the esophageal musculature,” and were most frequent at the start of recordings and during “conditions which are presumably unfavorable to the worm, such as trauma and irritation” (Roche et al., 1962). These authors did not speculate on the behavioral function that this esophageal behavior might serve.

### 3.4 Video analysis of esophageal behaviors in *A. ceylanicum* L4s

To correlate specific behaviors with different EPG waveforms, we made simultaneous EPG and video recordings of individual *A. ceylanicum* L4s. Observations below were based on slow-motion review of >2.5 h of video recordings of *A. ceylanicum* L4s in chips. Anatomical structures examined in the videos were (from anterior to posterior) the buccal capsule, esophagus, esophageal bulb (slight enlargement of the posterior esophagus), esophogeal-intestinal (EI) valve and the anteriormost region of the intestine (Nichols, 1956, Matsusaki et al., 1964, Matsusaki et al., 1965). The term ‘esophagus’ is used here synonomously with ‘pharynx.’

**Fig. 4:**
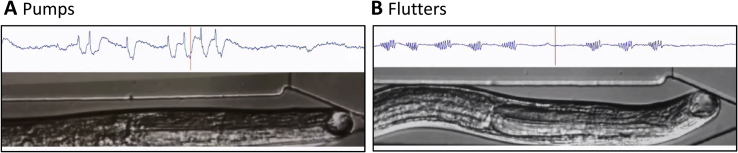
Simultaneous EPG and video analysis of esophageal (pharyngeal) behavior in an *A. ceylanicum* L4. The larva was oriented head-first in the worm trap (at right; see Fig. 1B), in RPMI with 20% CS, 20 °C. The EPG recordings scroll from left to right across the screen, with a vertical red line indicating the time corresponding to the video display. Video playback was slowed to 30% of original speed. The audio channel was synthesized from the EPG voltage signal (see 2.8). A. Esophageal (pharyngeal) pumping. B. The same worm, showing erratic contractions of the esophagus and EI valve openings, termed flutters. Observing the correspondence between EPG waveforms and esophageal behaviors is facilitated by listening to the audio track while watching the worm video. (For interpretation of the references to colour in this figure legend, the reader is referred to the web version of this article.)

Fig. 4 shows simultaneous video and EPG recordings from a representative *A. ceylanicum* L4. At rest, the esophageal lumen and EI valve were closed and the EPG recording was flat. As seen in Fig. 4A, characteristic pump waveforms in EPGs corresponded to esophageal pumps. Each pump was marked by dilation of the esophageal lumen (presumably via contraction of radial muscles surrounding the esophagus; Mapes, 1965, Brownlee et al., 1995b), which appeared synchronous along the length of the esophagus. Dilation was followed by closure of the lumen (presumably via relaxation of the radial muscles) and opening of the EI valve. In other parasitic nematodes, muscles fibers attach to the EI valve and there is innervation of the valve region (Mapes, 1965, Brownlee et al., 1995b), suggesting that valve openings may be neurally controlled rather than passive responses to increased intra-esophageal pressure. Neural control of the EI valve is also suggested by our observation of ‘twitches’ and valve openings in the absence of other esophageal movements (data not shown). In contrast to esophageal dilation, there was an anterior-to-posterior progression in lumenal closure, with the EI valve opening when closure reached the end of the esophagus. This sequence propelled esophageal contents posteriorly, through the EI valve into the intestine. An additional video of esophageal pumping and valve movements in an *A. ceylanicum* L4 is provided as Supplemental Content.

The following are the supplementary data related to this article:

Supplemental Content Video recording of esophageal pumping in an *A. ceylanicum* L4. The larva was oriented head-first in the worm trap (right), in RPMI with 20% CS, 20 °C. Video playback was slowed to 30% of original speed. Five pumps are shown, accompanied by coordinated opening and closing of the EI valve. Retrograde perfusate flow is present because perfusion had been turned off. Supplemental Content

2

In contrast to pumps, flutter EPG waveforms (Fig. 3Bii) corresponded to series of small, rapid contractions along the esophagus and repeated openings and closings of the EI valve (Fig. 4B), which prompted our use of the term ‘flutter’ for this behavior. The observed behavior thus aligns with the “uncoordinated inefficient” esophageal contractions reported in *A. caninum* adults by Roche et al. (1962). The small size and brief duration of esophageal dilations during flutters suggests that minimal suction was produced. In videos, hybrid EPG waveforms (Fig. 3Biii) clearly corresponded to pumps, with prominent dilation of the esophageal lumen, but accompanied by rapid, localized contractions of the esophagus. These behavioral observations support the classification of hybrid waveforms as pumps during data analysis (see 2.9).

It was possible to link some EPG voltage signals with esophageal movements. As expected, E and R spikes in pump waveforms corresponded to initiation and termination of esophageal dilation. Larger-amplitude pump waveforms were accompanied by more forceful esophageal contractions. The source of repetitive voltage deflections during flutters was less clear. The negative-going phase of deflections bore some resemblance to inhibitory postsynaptic potentials (IPSPs) produced in *C. elegans* pharyngeal muscles by motoneuron M3, via glutamate-gated chloride channels (Glu-Cls) (Franks et al., 2006). These IPSPs, which occur between E and R spikes, help terminate pharyngeal contraction. In this case, we would expect hybrid waveforms in *A. ceylanicum* L4s to have shorter durations than conventional pumps, but we did not observe this relationship (e.g., Fig. 3Bi and Biii; data not shown). Another possibility is that the voltage deflections during flutters reflected action potentials occurring asynchronously throughout esophageal muscles, corresponding to the localized contractions and valve openings. The relationship between voltage deflections and muscle contractions was most apparent when observing the esophagus while listening to the audio track of the EPG recording (Fig. 4B). The rapid voltage deflections during some pumps and all hybrid EPG waveforms (Fig. 3Bi and Biii) may likewise have resulted from localized contractions superimposed on lumenal dilation. The possible behavioral function of flutters is considered at the end of Section 3.6.

In previous experiments using the EPG platform, we relied exclusively on pharyngeal pumping as the readout of anthelmintic bioactivity. The presence of flutters in A. *ceylanicum* L4s raised the issue of whether flutters were as valid as pumps in quantifying effects of excitatory and inhibitory drugs on EPG activity. The presence of flutters also required that we modify the detection algorithm used to identify pumps in EPG recordings, to independently identify and count pumps and flutters. The latter was successfully accomplished (see 2.9) and we show below (see 3.6 and 3.7) that combining pumps and flutters into one category termed ‘EPG events’ produced the same results as counting pumps alone, while providing more statistical power.

### 3.5 Effects of perfusate-switching and 5HT on EPG activity in *A. ceylanicum* L4s

**Fig. 5:**
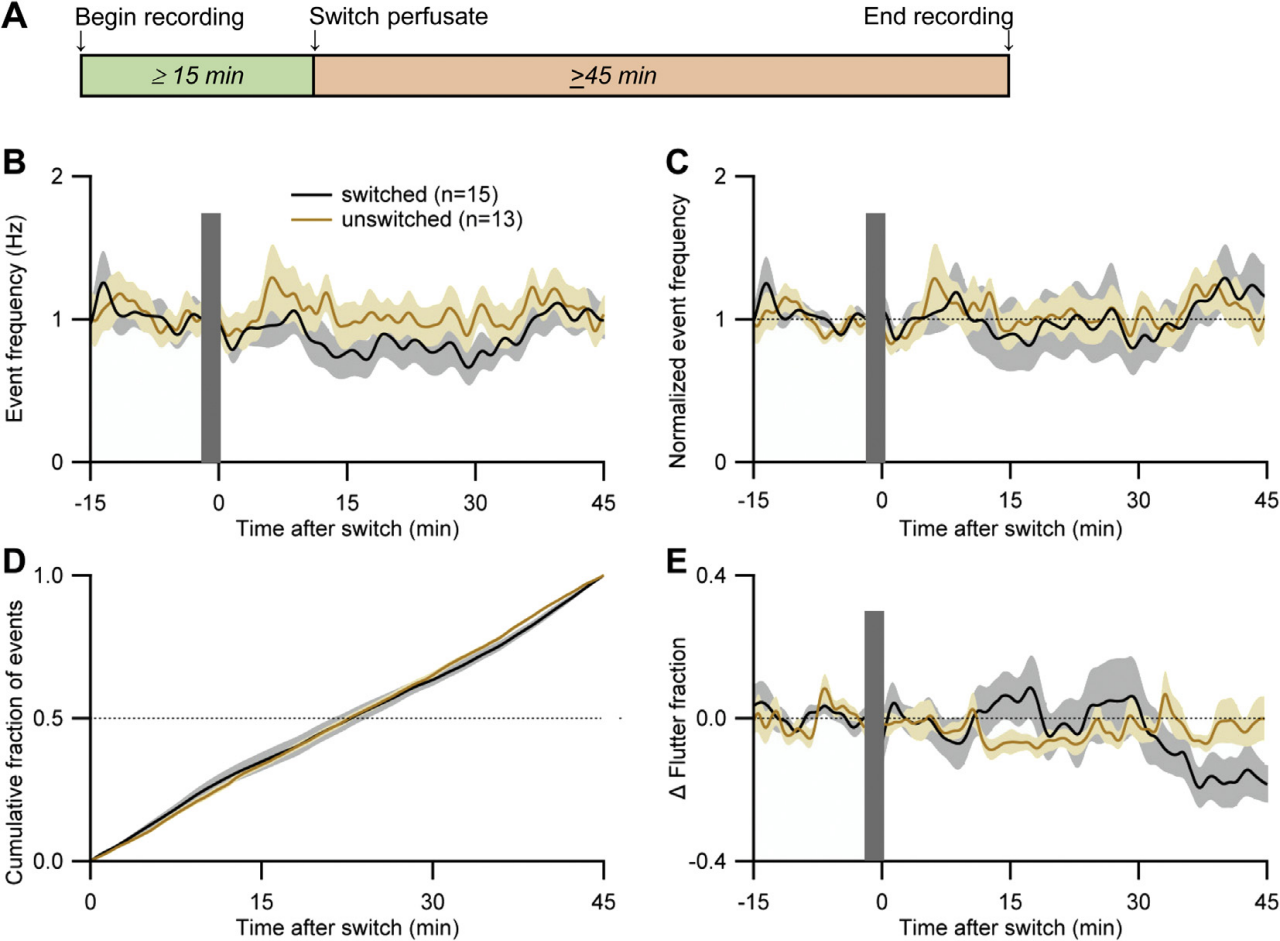
EPG activity in *A. ceylanicum* L4s under control conditions. A. Protocol for drug perfusion experiments. B, C. EPG activity during perfusion with control medium (RPMI-c with 20% CS) with (brown) or without (black) switching the perfusate to the same medium at *t* = 0. Grey bars mask the electrical artifact produced by switching perfusate in the switched group. Pump and flutter counts were combined and jointly termed EPG ‘events.’ Raw data in B; normalized (see 2.9) data in C (dotted line marks normalized frequency of 1.0). Lines and shading show mean ± S.E.M. in all panels; *n* (number of worms) shown in key. D. Cumulative fraction of events (see 2.9) for 45 min after the time of the switch in the switched group, or corresponding time in the unswitched group. *CF*_*50*_ values (the time at which 50% of the total number of events had occurred) for worms in the switched and unswitched groups did not differ significantly (*P* > 0.3; 2-tailed Wilcoxon Mann-Whitney U test). E. Change (Δ) in the ‘flutter fraction,’ (the proportion of EPG events that were flutters), expressed as the deviation from each worm's baseline flutter fraction (see 2.9); baseline flutter fraction was between 0.006 and 0.8 in both groups (not shown). Dotted line marks zero change in flutter fraction. Same data set in B–E. All recordings at 32–37 °C. (For interpretation of the references to colour in this figure legend, the reader is referred to the web version of this article.)

Fig. 5A shows the protocol used for experiments with *A. ceylanicum* L4s, which includes switching perfusate in mid-experiment. During the 1-to-2 min interruption of perfusate flow during the switch, worms drifted backward from the worm trap (Fig. 1B) and were propelled back into the trap after flow was re-established. Accordingly, we first tested whether the mechanical disturbance caused by switching perfusate disturbed EPG activity.

Fig. 5B and C shows the frequency of EPG events over time in two groups of L4s. One group (brown) was perfused with control medium without interruption while the other group (black) was switched from control medium to the same medium at *t* = 0. Raw data in Fig. 5B show that event frequency remained steady at ∼0.8–1 Hz in both groups. The same data are shown normalized in Fig. 5C to compensate for minor variations between worms in baseline event frequency; only normalized data are shown in subsequent Figures. Fig. 5D compares the cumulative fraction of events occurring after the time of the switch (*t* = 0), with no significant difference between the two groups at *CF*_*50*_ (our standard index of comparison). Fig. 5E shows the proportion of events that were flutters (‘flutter fraction’; see 2.9; baseline flutter fractions are provided in Figure Legends) during the experiment. Flutter fraction remained relatively stable in both groups including in the switched group; this group showed a decline in flutter fraction starting ∼30 min post-switch, for unknown reasons presumably unrelated to the switch. When flutters were omitted from the *CF*_*50*_ analysis, results similar to those in Fig. 5D were obtained (data not shown).

In summary, the mechanical disturbance cause by switching perfusate perturbed neither EPG event frequency nor the flutter fraction in *A. ceylanicum* L4s.

**Fig. 6:**
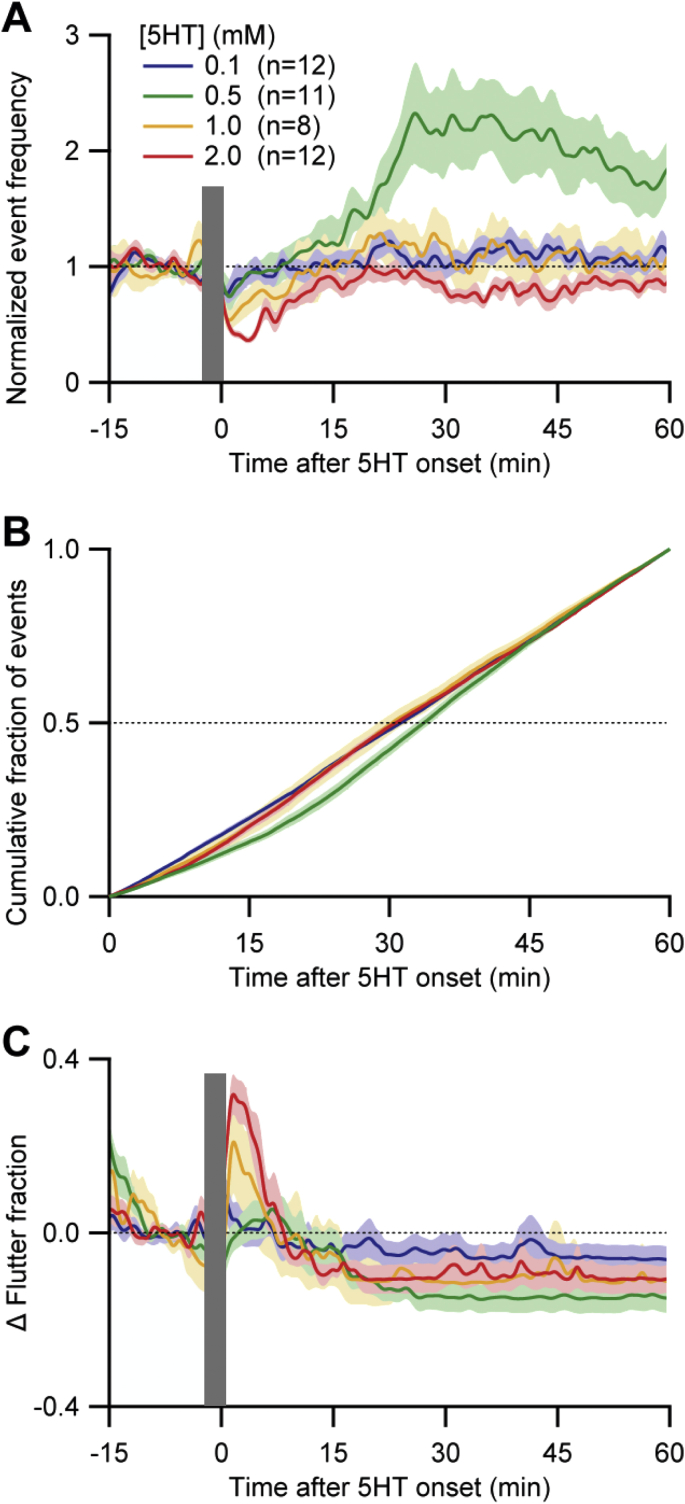
Effect of 5HT on EPG activity in *A. ceylanicum* L4s. A. Dose-response relationship of normalized event frequency (pumps + flutters) versus 5HT concentration. At *t* = 0 min, perfusate was switched (grey bar) from control medium (RPMI-c with 20% CS) to the same medium with different concentrations of 5HT (see key). Lines and shading show mean ± S.E.M.; *n* (number of worms) shown in key. Dotted line marks normalized frequency of 1.0. B. Cumulative fraction of events for 60 min after switching to 5HT-containing perfusate. *CF*_*50*_ values (dotted line) did not differ significantly between groups except for the 0.5 mM 5HT group, which differed from all other groups (*P* < 0.04; 2-tailed Wilcoxon Mann-Whitney U test). C. Change (Δ) in ‘flutter fraction,’ (the proportion of total EPG events that were flutters); baseline flutter fraction was between 0.05 and 0.2 in all groups (not shown). Dotted line marks zero change in flutter fraction. Same data set in A–C. All recordings at 34–36 °C.

Although *A. ceylanicum* L4s produced sustained EPG activity in the absence of 5HT, we tested whether activity was enhanced by 5HT. 5HT stimulates pumping in *C. elegans* and other free-living and parasitic nematodes (Brownlee et al., 1995a, Tahseen et al., 2003, Chiang et al., 2006, Song and Avery, 2012), with a wide effective range of ∼0.005–20 mM reported in intact worms. Fig. 6A shows the effects of 5HT on normalized EPG event frequency in *A. ceylanicum* L4s. Switching the perfusate to 5HT-containing medium had several effects. First, there was a transient decrease in EPG activity immediately following the switch, which was most pronounced in the highest-concentration group (2 mM 5HT); based on Fig. 5, this inhibition resulted from the 5HT and not the switch. By ∼10–15 min post-switch, EPG activity recovered to near baseline levels in all groups. The most striking effect of 5HT was an increase (initially > 2-fold) in EPG event frequency in the 0.5 mM 5HT group, which peaked by ∼25 min and then waned (Fig. 6A). The 15–25 min latency for 5HT to exert maximal effect on EPG event frequency is similar to that in *C. elegans* (unpublished data). *CF*_*50*_ values did not differ significantly among treatment groups, except for the 0.5 mM 5HT group (Fig. 6B). Results similar to those in Fig. 6 were obtained when only pumps were analyzed (data not shown). Fig. 6A and B thus demonstrate a U-shaped concentration dependence of 5HT's effect on EPG event frequency, with peak effectiveness (among the tested concentrations) at 0.5 mM 5HT.

Fig. 6C presents flutter fraction data for the different 5HT groups. In the two highest concentration groups (1 and 2 mM 5HT), the switch to 5HT caused a rapid but transient increase in flutter fraction, which recovered to near baseline levels after ∼10 min. Subsequently, flutter fraction was generally reduced from baseline levels for the remainder of the experiment, in all groups (Fig. 6C). In summary, 0.5 mM 5HT, but not higher or lower concentrations, increased EPG activity in *A. ceylanicum* L4s.

### 3.6 Inhibition of EPG activity by IVM in *A. ceylanicum* L4s

For human hookworm infection, the most efficacious anthelmintics are albendazole and mebendazole (Keiser and Utzinger, 2010) but IVM also kills *A. ceylanicum* (Behnke et al., 1993, Richards et al., 1995, Y. Hu et al., 2013). IVM was originally developed as a veterinary anthelmintic and is still used as such (Geary, 2005); acting on Glu-Cls, it causes paralysis and death of intestinal nematodes (Wolstenholme and Rogers, 2005). We tested IVM on *A. ceylanicum* L4s rather than a benzimidazole because IVM terminates EPG activity in *C. elegans* (Lockery et al., 2012) and a concern that benzimidazoles’ mode of action (microtubule destabilization) may be too slow to produce an EPG phenotype during a 45–60 min recording.

**Fig. 7:**
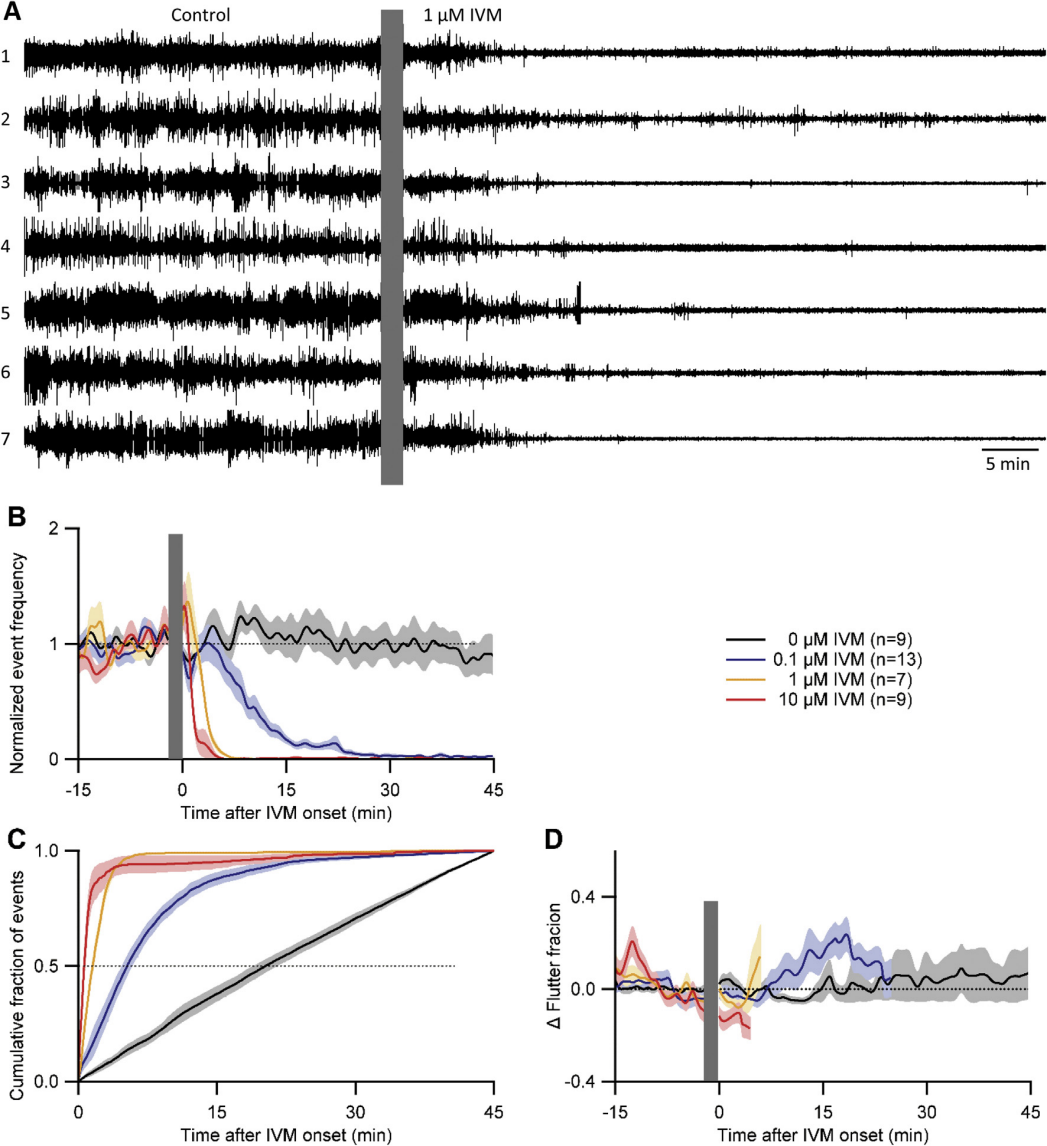
Effect of IVM on EPG activity in L4 *A. ceylanicum*. A. Simultaneous EPG recordings from seven worms (numbered 1 to 7) in one chip: each trace from a different worm. Control activity was recorded in RPMI-c with 20% CS, followed by a switch (grey bar) to the same medium with 1 μM IVM. B. Dose-response relationship of normalized event (pumps + flutters) frequency versus IVM concentration. Lines and shading show mean ± S.E.M.; *n* (number of worms) shown in key. Dotted line marks normalized frequency of 1.0. C. Cumulative fraction of events for 45 min after switching to IVM-containing perfusate. *CF*_*50*_ values (dotted line) differed significantly between all groups (*P* < 0.004; 2-tailed Wilcoxon Mann-Whitney U-tests). D. Change (Δ) in ‘flutter fraction,’ (the proportion of total EPG events that were flutters); baseline flutter fraction was between 0.05 and 0.2 in all groups (not shown). Lines were terminated when EPG activity ceased (1 μM and 10 μM IVM groups). Dotted line marks zero change in flutter fraction. Same data set in B–D. All recordings at 34–36 °C.

Fig. 7A displays representative recordings in *A. ceylanicum* L4s, showing that EPG activity was terminated by switching the perfusate to 1 μM IVM. Ivermectin caused EPG signals to decrease in amplitude, as also seen in *C. elegans* (Lockery et al., 2012). Most activity ceased by 10–15 min after IVM onset. Fig. 7B demonstrates that this inhibition was concentration-dependent. In control larvae, normalized EPG event frequency was stable for the duration of the experiment. The two highest concentrations of IVM tested (1 and 10 μM) rapidly terminated EPG activity whereas, in 0.1 μM IVM, activity continued for ∼30 min. *CF*_*50*_ values differed significantly between all of the groups (Fig. 7C) and similar results were obtained when only pumps were counted (data not shown). Fig. 7D shows that flutter fraction was stable in the control group and that the IVM groups showed no consistent pattern after the switch. Notably, IVM did not cause a sudden increase in flutter fraction, as seen for the high-concentration 5HT groups (Fig. 6C). Thus, IVM experiments did not provide additional insight into whether flutters represent aversive responses (Roche et al., 1962).

The micromolar concentrations of IVM used here and in *A. suum* experiments (see 3.8) are higher than needed to kill intestinal worms *in vivo* (Behnke et al., 1993) and hence might be considered ‘unphysiological.’ In the case of *A. ceylanicum*, Richards et al. (1995) discuss why higher concentrations are needed *in vitro*, including the desire for rapid endpoints (e.g., cessation of pharyngeal activity within 30 min; Fig. 7B) as opposed to the days to weeks of lower-dose exposure that cause *in vivo* endpoints such as reduced egg production or worm expulsion.

In *C. elegans*, 5HT influences pharyngeal behavior via multiple neural pathways and 5HT receptor subtypes (Song and Avery, 2012, Trojanowski et al., 2016). Comparable information is not available for hookworms. The details of 5HT's effects in *A. ceylanicum* remain to be determined, but the enhancement of EPG activity by 5HT treatment resembles that seen in other nematodes. The finding that *A. ceylanicum* L4s generated sustained EPG activity without 5HT in the medium is advantageous for experiments. 5HT treatment is an unnatural stimulus that overrides normal feeding circuitry and, by driving supra-normal EPG activity, could potentially decrease sensitivity to anthelmintic compounds. Blood serum in the medium (20% CS in RPMI-c) apparently provided a sufficient feeding-inducing stimulus in *A. ceylanicum* L4s, without exogenous 5HT (Roche et al., 1971, Hawdon and Schad, 1990). A minor caveat is that sera may contain low levels of 5HT (Mothersill et al., 2010). The ability of a natural food stimulus to drive pharyngeal activity is also seen in *C. elegans*, a bacteriovore, in which perfusing edible bacteria through an EPG chip evokes sustained pharyngeal pumping, but at a lower frequency than with 10 mM 5HT (unpublished data). Thus, in both *A. ceylanicum* and *C. elegans*, maximal pharyngeal pump frequencies are achieved with 5HT treatment rather than natural feeding stimuli.

These experiments with 5HT and IVM provide some potential insight into the behavioral significance of flutters. Depending on feeding habit, nematodes show a variety of esophageal/pharyngeal behaviors (e.g., Chiang et al., 2006, Bhatla et al., 2015, Wilecki et al., 2015). However, other than *A. ceylanicum* L4s (current study), and *A. caninum* adults (Roche et al., 1962), flutter waveforms and behaviors have not, to our knowledge, been reported. It is thus unknown how widespread this behavior is. Roche et al. (1962) proposed that flutters are responses to aversive conditions; in the present experiments, an increase in flutter fraction might reveal such a response. The only instance in which flutter fraction increased after switching perfusate was for the two highest concentrations of 5HT (Fig. 5, Fig. 6, Fig. 7D). In *C. elegans*, 5HT increases sensitivity to aversive stimuli (Chao et al., 2004), consistent with a potentially aversive response in *A. ceylanicum*. It is perhaps surprising that IVM did not evoke a similar response, because of its toxicity to nematodes. One difficulty in interpreting responses to 5HT and IVM is that, as drugs that perturb electrophysiological signaling, they may activate behaviors inappropriately. Thus, the increase in flutter fraction caused by 5HT may be irrelevant to normal behavior. Clearer results would be obtained by recording EPGs while presenting putatively aversive stimuli with more physiological relevance, such as low pH, high temperature or physical injury.

Nevertheless, some potential functions of flutters can be considered. Unlike pumps, flutters produced little esophageal dilation, suggesting that they were relatively ineffective in sucking blood or tissue into the buccal cavity or esophagus. During an endoscopic study of hookworms feeding in the human intestine, Barakat et al. (2012) described three behaviors: “… (1) the mucosal piercing process, which is a mechanical, quickly spinning, body-pushing movement that caused piercing within a few seconds …; (2) repeated feeding by the same worm within a short time …; (3) graceful smooth movement after fixation of the worm to the mucosa, which was maintained during the rest of the meal …”. While positioned in microchannels, *A. ceylanicum* L4s often produced sinuous body movements, lengthening and shortening of the body, and sometimes spinning movements, which may have some relationship to behaviors expressed *in vivo*. Based on visual review of video recordings of *A. ceylanicum* L4s in chips, we did not detect a reliable correspondence between pumps, flutters and any whole-body movements, but a definitive conclusion would require more detailed analysis. An intriguing possibility is that flutters are associated with the mucosal piercing process, a behavior specially adapted for blood feeders.

Another potential function of flutters relates to esophageal secretion. Several prominent secretory glands empty into the hookworm esophagus and buccal cavity (Eiff, 1966; Smith, 1976). The excretory/secretary products of hookworms include diverse anticoagulants, immunomodulatory agents, proteases and other molecules (Mulvenna et al., 2009), with both anticoagulants and digestive enzymes being present specifically in esophageal glands (Feng et al., 2007, Jiang et al., 2011). In an excellent description of feeding behavior *in vivo*, Kalkofen (1970) reported that *A. caninum* adults suck a large bolus of host tissue into the buccal capsule every 6–15 min, which is digested and sucked into the esophagus and intestine. Potentially, esophageal contractions during flutters could help release digestive enzymes and anticoagulants and/or mix them with ingesta. Esophageal flutters and associated EI valve openings might also permit the retrograde flow of digestive enzymes from the intestine to the esophagus. To resolve such questions, movements of esophageal and intestinal contents could be visualized by introducing fluorescent microspheres or oil droplets into the medium (Kiyama et al., 2012, Bhatla et al., 2015).

### 3.7 EPG recordings in *Ascaris suum* L3s

We next tested *A. suum* L3s, obtained from pig lungs 7 d after infection (see 2.4), in the microfluidic EPG platform. This stage was selected because it required only minor modification of channel size in the microfluidic EPG chips (see 2.5). Our access to *A. suum* larvae was limited, so experiments were less comprehensive than for *A. ceylanicum*.

**Fig. 8:**
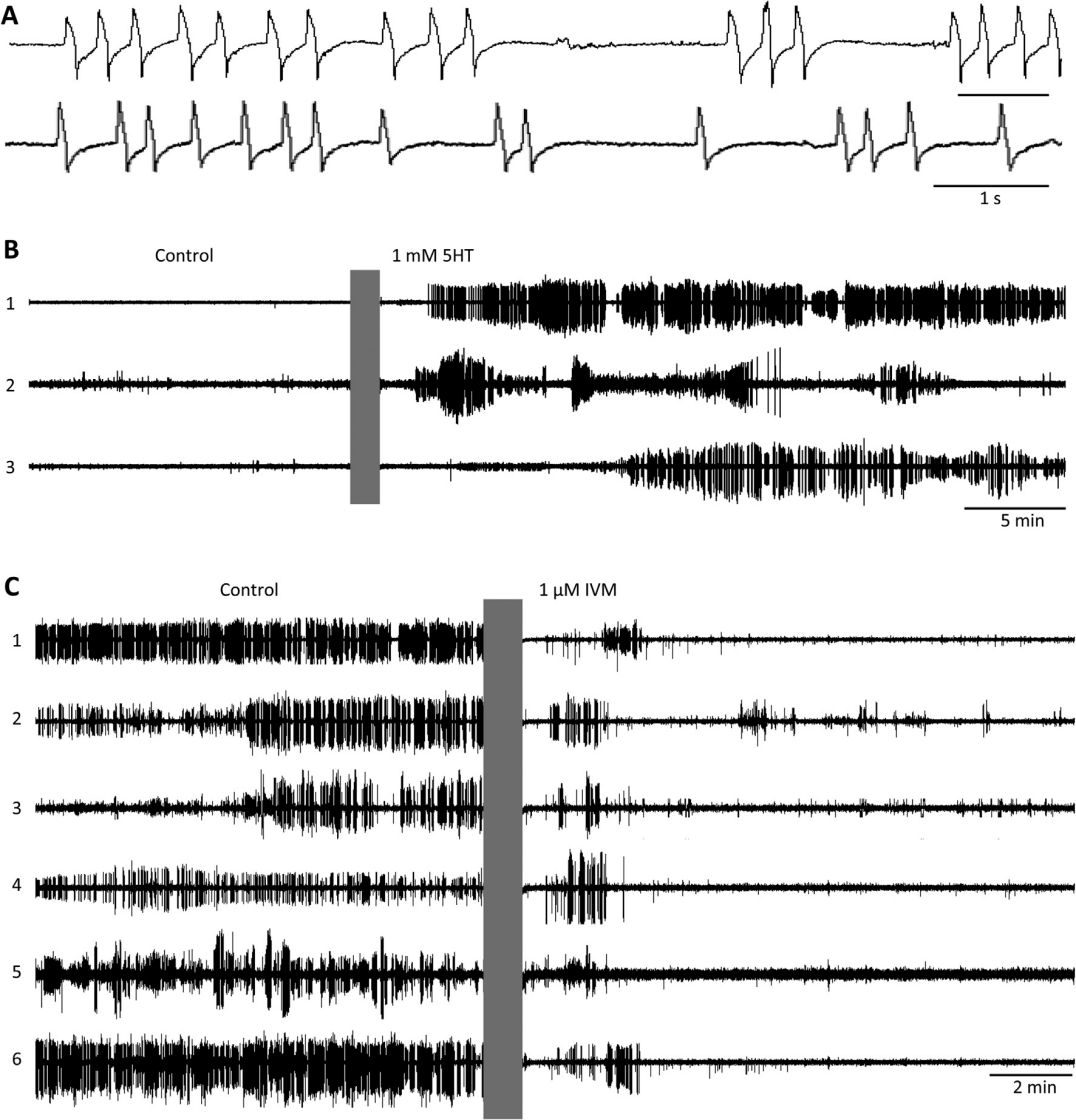
EPG recordings from *A. suum* L3s. A. Representative EPG recordings during pharyngeal pumping by two *A. suum* L3s in different chips, in RPMI-c with 10% CaS and 1 mM 5HT. B. Induction of pharyngeal pumping by 5HT. Simultaneous EPG recordings from 3 worms (numbered 1 to 3) in one chip: each trace from a different worm. Control period was recorded in RPMI-c with 10% CaS, followed by a switch (grey bar) to the same medium with 1 mM 5HT. C. Simultaneous EPG recordings from six worms (numbered 1 to 6) in one chip: each trace from a different worm. Control activity was recorded in RPMI-c with 10% CaS and 1 mM 5HT, followed by a switch (grey bar) to the same medium with 1 μM IVM. All recordings at 37–38 °C.

Fig. 8A shows representative EPG waveforms recorded in *A. suum* L3s. The waveforms were characteristic of pharyngeal pumping in other nematodes (see 3.2) and visual observation confirmed their correspondence to pharyngeal pumps (data not shown). No flutter waveforms or behaviors were observed. Table 1 shows pump amplitude and duration data for *A. suum* L3s.

*A. suum* exhibits 5HT immunoreactivity and has multiple 5HT receptor isoforms (Johnson et al., 1996, Huang et al., 2002); in the absence of 5HT, pharyngeal preparations do not exhibit spontaneous pumping, but do so in the presence of 10–1000 μM 5HT (Brownlee et al., 1995a, Brownlee et al., 1997). Fig. 8B shows the effect of 1 mM 5HT on *A. suum* L3s; there was little or no EPG activity under control conditions whereas 5HT evoked sustained pumping. The onset of robust pumping was relatively rapid (∼5–15 min).

The preferred treatment for human ascariasis is albendazole, with mebendazole and pyrantel as alternatives (Keiser and Utzinger, 2008). *In vitro*, IVM has been shown to kill or inhibit pharyngeal pumping in *A. suum* (Brownlee et al., 1997, Dmitryjuk et al., 2014) and, in pigs, it is effective against both larval and adult *A. suum* (Borgsteede et al., 2007). Fig. 8C shows the effect of IVM on pumping in *A. suum* L3s. During the baseline period, in 1 mM 5HT, mean pump frequency was 1.16 ± 0.20 Hz (S.E.M; *n* = 6 worms). For comparison (Brownlee et al., 1995a), reported a mean pump frequency of 0.5 Hz in *A. suum* adults in 100 μM 5HT, at a temperature similar to our recordings. In Fig. 8C, switching the perfusate to 1 μM IVM caused most pumping to cease within 3–4 min. Brownlee et al. (1997) reported that 1 μM IVM inhibited pumping as rapidly as 60 s after application, in dissected adult *A. suum* in which drugs were applied directly to the pharynx.

Because of the limited number of larvae available, we did not generate concentration-response curves for 5HT and IVM on *A. suum* L3s. Nevertheless, these experiments identified conditions under which *A. suum* produced robust pharyngeal pumping in microfluidic EPG chips and we replicated previous findings in adult *A. suum* regarding effects of 5HT and IVM on pharyngeal pumping. Future experiments can now investigate these effects in detail, including potential differences in pharyngeal physiology and anthelmintic sensitivity between larvae and adult worms.

## 4 Conclusions

Technological advances often exert outsized influence on the progress of scientific research, as seen in the case of high-throughput screening for anthelmintic drug candidates (e.g., Buckingham et al., 2014, Bulman et al., 2015, Burns et al., 2015). The present study addresses another key aspect of the screening process: secondary screening to prioritize hits and investigate mode of action. Our work is motivated by the urgent need to develop new anthelmintic treatments for humans and animals, and the importance of neurotransmitter receptors and ion channels as potential drug targets. The 8-channel microfluidic EPG chip provides a convenient and powerful new tool for detecting the integrity of electrophysiological signaling in nematodes and its perturbation by applied drugs, compounds or natural products. The throughput of the current 8-channel EPG chip can be increased by increasing the number of recording modules per chip, or running multiple chips in parallel, but is unlikely to approach the throughput of large, automated screening platforms. Instead, EPG analysis can help prioritize hits and assist in determining mode of action: e.g., if used in conjunction with *C. elegans* mutants or molecular tools such as transgenesis and RNA interference that are increasingly available in parasitic nematodes (Ward, 2015). Alternatively, when supplies of parasitic worms are limited and/or drug candidates are expected to have rapid electrophysiological actions, the 8-channel microfluidic EPG platform could provide a useful primary screen. The experimental design of recording EPGs from individual worms before and during exposure to a drug (illustrated in Fig. 7, Fig. 8C) provides a powerful advantage of within-subjects statistical analysis.

Ultimately, promising anthelmintic compounds must be tested on the actual species being targeted for control; to advance this capability, we validated the EPG platform in two STH species relevant to human health in low-resource regions of the world. Specifically, we found that under suitable conditions, host-stage larvae of *A. ceylanicum* and *A. suum* produce robust, sustained EPG activity in microfluidic chips, allowing stimulatory (Fig. 6, Fig. 8B) or inhibitory (Fig. 7, Fig. 8C) drugs to be readily detected. In contrast to less specific readouts of anthelmintic activity such as development, motility or death, EPG recordings can provide more direct access to underlying mechanisms. Furthermore, the ability to rapidly record thousands of EPG waveforms from individual worms provides exceptional statistical power. This capability may be valuable in detecting drug-resistant phenotypes, or for distinguishing different species within nematode populations. To permit the separation of worms based on their EPG phenotype, NemaMetrix (www.nemametrix.com) is developing a ‘sorting chip’ in which individual worms can be sorted into separate chambers for discard or recovery (e.g., sorting susceptible and resistant worms based on pharyngeal pumping that persists in the presence of an anthelmintic drug).

In experiments to be published elsewhere, we have used the microfluidic EPG platform to screen a library for anthelmintic candidates; demonstrate anthelmintic activity in a natural product used traditionally as a vermifuge; and investigated *C. elegans* models of human aging and disease. We are also optimizing chip design and experimental conditions for microfluidic EPG recordings from additional species of parasitic and free-living nematodes. We recommend this new technology as a versatile addition to the experimental toolbox for anthelmintic drug development and studies of drug resistance, basic research on nematode feeding behavior, and other applications in which an electrophysiological readout can provide unique insights into nematode biology.

## Conflicts of interest

The authors declare the following potential competing financial interests: JCW, KJR, SRL and WMR own equity in NemaMetrix, Inc., which holds the sole commercial license for the microfluidic EPG device reported here. A patent application from University of Oregon is pending, with SRL as the inventor.

## Author contributions

JCW, JMH, JJV, JFU and KJR designed the experiments. KJR and MK performed the experiments. WMR and JCW performed the data analysis. SRL contributed to chip design. JCW drafted the manuscript. All authors participated in editing the manuscript.

## Figures and Tables

**Table 1:** Properties of EPG waveforms during pharyngeal pumping.

Species and stage	Pump amplitude (μV)	Pump duration (ms)
*A. caninum* aL3	60 ± 5^a,c^	62 ± 5
*A. ceylanicum* aL3	65 ± 10^a,c^	139 ± 14^d^
*A. ceylanicum* L4	224 ± 45^b^	109 ± 10^d^
*A. suum* L3	178 ± 16^b^	73 ± 3

Values are mean ± S.E.M., *n* = 14 worms/group except *A. ceylanicum* aL3, *n* = 8. Mean number of pumps analyzed per worm was 306 ± 48 pumps (S.E.M., *n* = 4 groups), recorded under control conditions. Most *A. ceylanicum* aL3 recordings were made at room temperature; all other groups were recorded at ∼34–38 °C. Pump amplitude was measured peak-to-peak (E to R spike); ^a,b^ members of these pairs did not differ significantly (*P* ≥ 0.87); ^c^ both aL3 groups differed from the other groups (*P* < 0.001). Pump duration was measured as the E to R interval; ^d^ the two *A. ceylanicum* groups did not differ (*P* = 0.17) whereas all other groups differed significantly (*P* ≤ 0.024). Two-tailed Wilcoxon Mann-Whitney U-tests.
