# Supplementary figures and images for: Microfluidic platform for electrophysiological recordings from host-stage hookworm and Ascaris suum larvae: A new tool for anthelmintic research
Source: Int J Parasitol Drugs Drug Resist. 2016 Sep 15;6(3):314–28. doi: 10.1016/j.ijpddr.2016.08.001 (PMC5196495; doi:10.1016/j.ijpddr.2016.08.001)

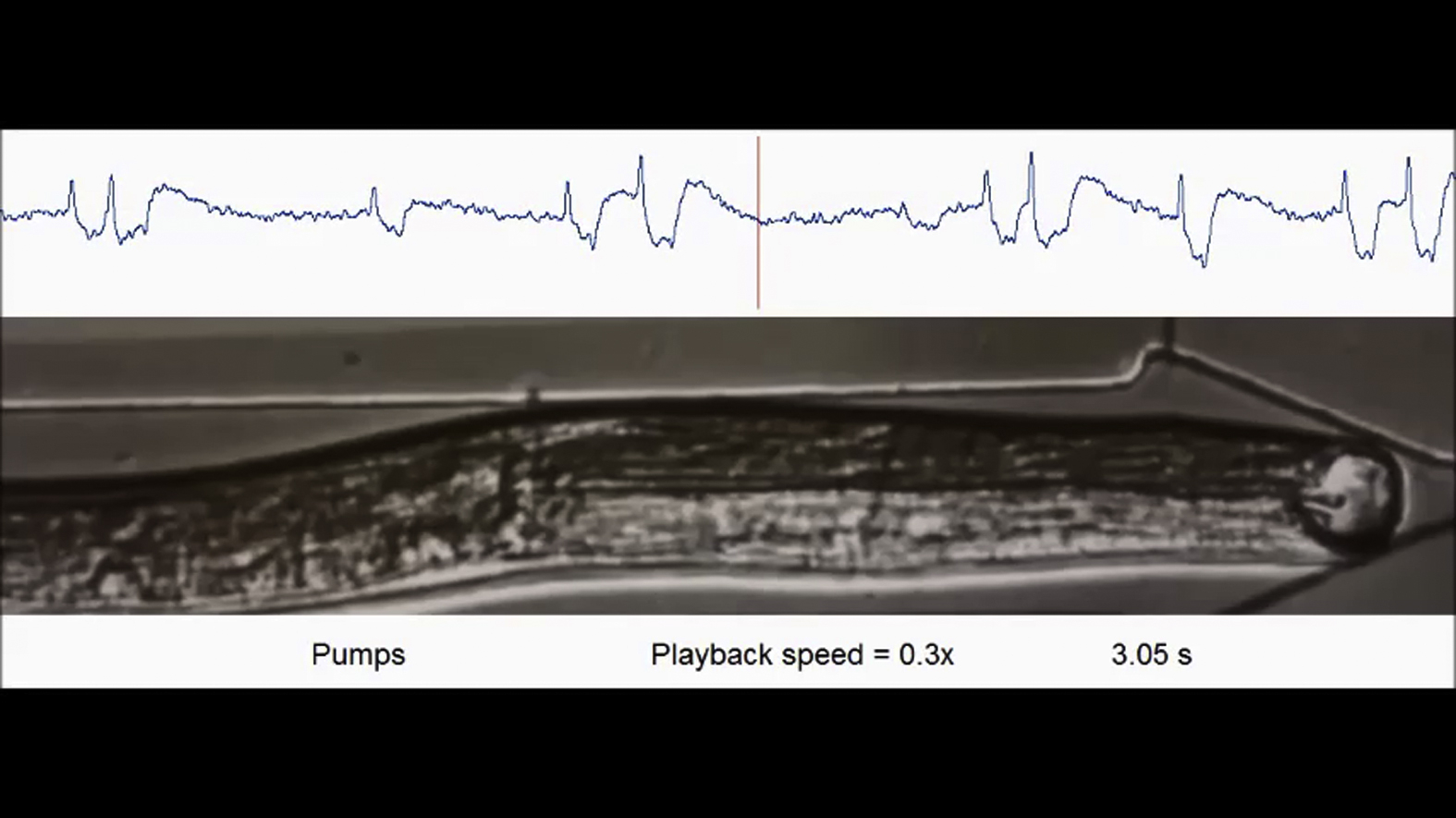

Supplement: Supplemental Content — Video recording of esophageal pumping in an A. ceylanicum L4. The larva was oriented head-first in the worm trap (right), in RPMI with 20% CS, 20 °C. Video playback was slowed to 30% of original speed. Five pumps are shown, accompanied by coordinated opening and closing of the EI valve. Retrograde perfusate flow is present because perfusion had been turned off. [file mmc1.jpg]

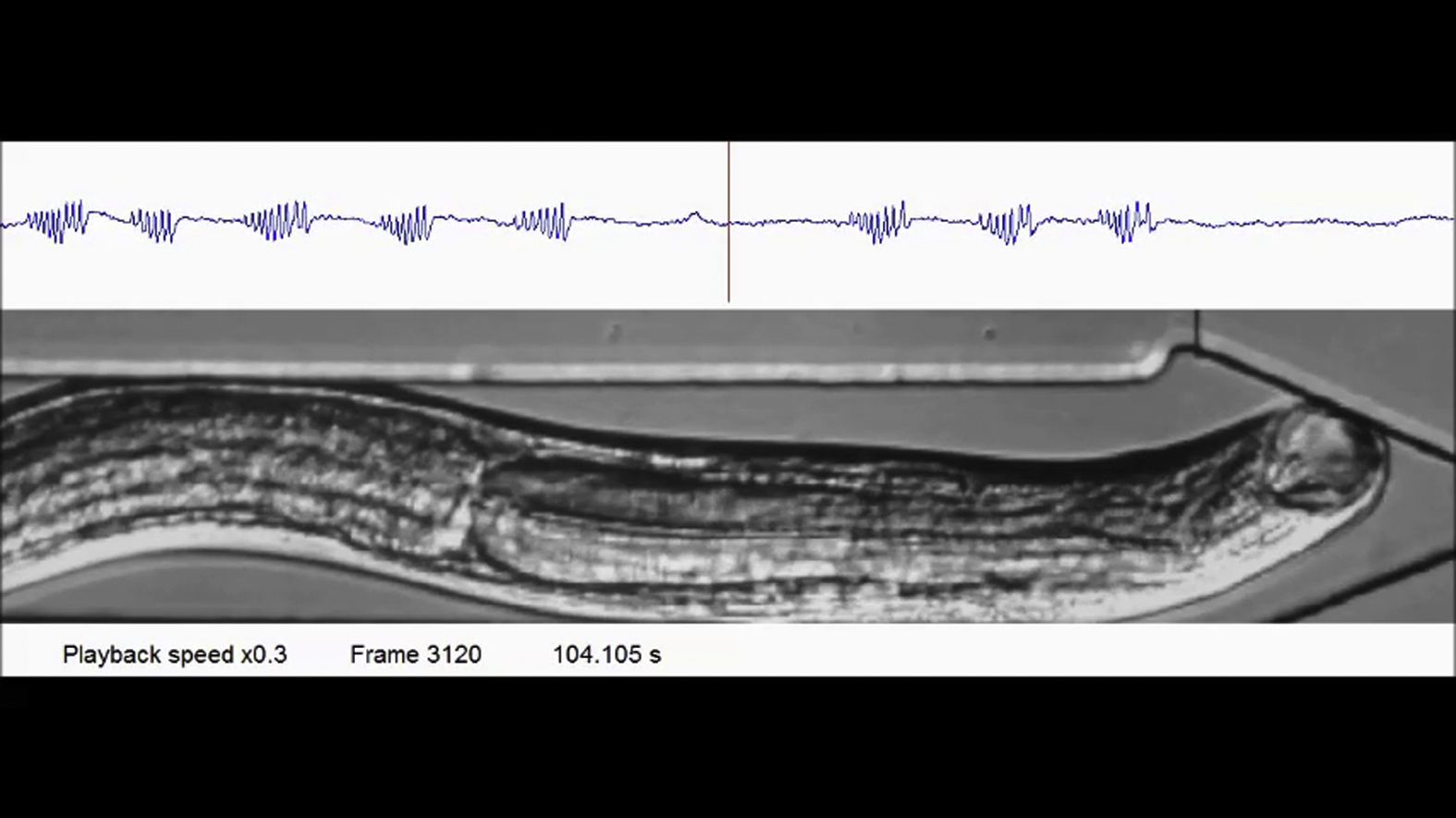

Supplement: Supplementary file 2 — 2 [file mmc2.jpg]

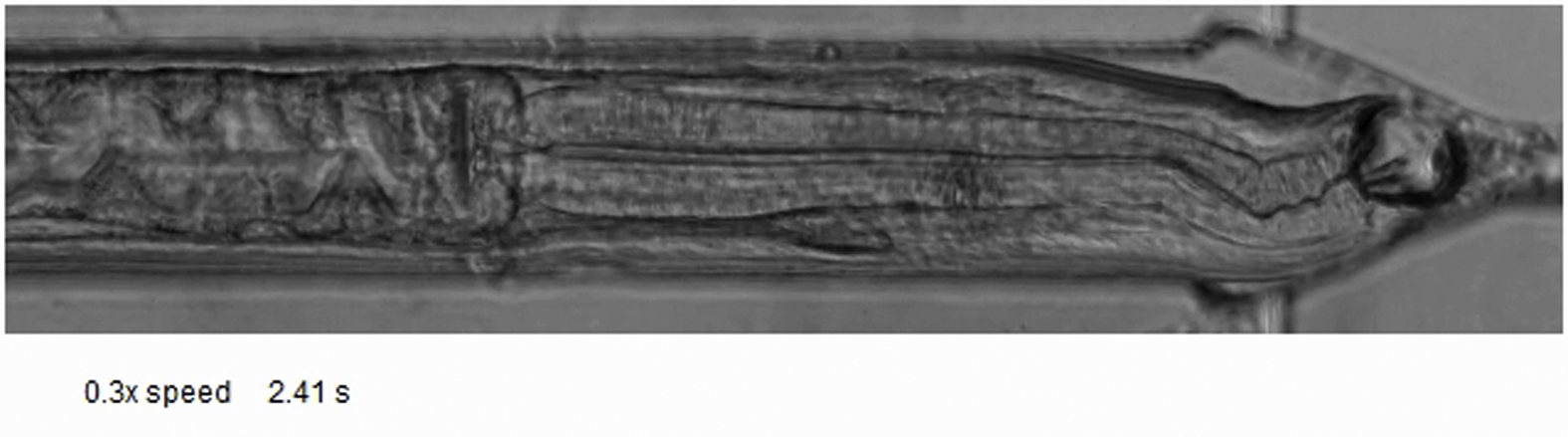

Supplement: Supplementary file 3 [file mmc3.jpg]
